# Relationship between all-cause mortality and triglyceride-glucose-body mass index in elderly patients with intravenous thrombolysis for acute ischemic stroke: a retrospective cohort study

**DOI:** 10.3389/fneur.2025.1689313

**Published:** 2026-01-05

**Authors:** Yuan Cheng, Mingfeng Zhai, Zhubiao Xie

**Affiliations:** 1 Fuyang Medical College, Fuyang Normal University, Fuyang, China; 2 Department of Neurology, The Affiliated Fuyang People’s Hospital of Anhui Medical University, Fuyang, China; 3 Intensive Care Unit, The Second Hospital Affiliated to Fuyang Normal University, Fuyang, China

**Keywords:** acute ischemic stroke, all-cause mortality, triglyceride-glucose-body mass index, thrombolysis, insulin resistance

## Abstract

**Background:**

Triglyceride-glucose-body mass index (TyG-BMI) has been shown to be a reliable surrogate for insulin resistance (IR), but the relationship between TyG-BMI and acute ischemic stroke (AIS) is unclear. In this study, we investigated the relationship between TyG-BMI and long-term all-cause mortality in elderly patients with intravenous thrombolysis for AIS.

**Methods:**

We enrolled 452 elderly patients with acute ischemic stroke treated with intravenous thrombolysis with alteplase and divided them into four groups according to TyG-BMI quartiles, and the endpoint event of the study was all-cause death from AIS. The Kaplan–Meier (K-M) curve method was used to compare the outcomes among the groups, while multivariate Cox proportional risk regression models and restricted cubic spline (RCS) were utilized to explore the association between TyG-BMI and these outcomes. In addition, subgroup analyses were performed.

**Results:**

A total of 452 elderly patients with intravenous thrombolysis for AIS were included, with a median age of 73.0 years, an interquartile range of 68.0–78.0 years, 58.4% males, a mean TyG-BMI of 205.54 ± 38.51. The follow-up period was more than six years, after which 75 (16.6%) patients died. K-M curve analysis demonstrated that patients with lower TyG-BMI levels had a higher risk of long-term all-cause mortality from AIS. When TyG-BMI was classified according to quartiles, Cox proportional risk regression analysis confirmed that both the Q1 group [HR, 3.482; 95% CI: 1.560–7.774; *p* = 0.002], Q3 group [HR,2.819; 95% CI: 1.196–6.640; *p* = 0.018] and the Q4 group [HR, 2.928; 95% CI: 1.259–6.806; *p* = 0.013] were associated with higher all-cause mortality rates, using the quartile with the lowest mortality as the reference. In addition, restricted cubic spline curves revealed a nonlinear relationship between TyG-BMI and long-term all-cause mortality (nonlinear *p*-value = 0.048).

**Conclusion:**

In this study, we found a U-shaped correlation between TyG-BMI and long-term all-cause mortality in elderly patients with AIS undergoing intravenous thrombolysis. TyG-BMI can be used as a predictor of all-cause mortality in this group of patients.

## Introduction

Acute ischemic stroke (AIS) is ischemia and necrosis of brain tissue due to a sudden interruption of blood flow to the brain, which causes a major global burden of disease due to its high morbidity, disability, and mortality rates (1, 2). Clinical recombinant tissue-type plasminogen activator (rt-PA), a first-line clinical agent for the treatment of AIS, can rapidly restore cerebral blood flow by activating plasminogen to dissolve thrombus formation (3, 4). This is beneficial in improving the clinical prognosis of patients with AIS, but it has a limited therapeutic time window, a risk of ischemia–reperfusion injury, stroke recurrence, some patients experiencing symptomatic intracranial hemorrhage, and severe neurological deficits left behind in as many as 1/3 of patients (5–7). Therefore, it is of great practical importance to identify biomarkers that can accurately predict the prognosis of patients with intravenous thrombolysis in AIS.

Insulin resistance (IR), as a central pathophysiological mechanism of several metabolic diseases (type 1 diabetes mellitus, metabolic syndrome, obesity, polycystic ovary syndrome, etc.) (8–10), refers to the decrease in the body’s sensitivity to insulin, which leads to ineffective glucose uptake and utilization by the target tissues and cells (11). Previous studies have shown that IR contributes to the poor prognosis of stroke through multiple inflammatory or metabolic pathways, and it has been shown to be an emerging risk factor for stroke (12). In recent years, the triglyceride-glucose-body mass index (TyG-BMI) is replacing the high insulin-normal glucose clamp test and the homeostasis model assessment of insulin resistance (HOMA-IR) as a sensitive indicator for IR detection due to its convenience, noninvasiveness, and ease of measurement (13–15).

Studies have shown that TyG-BMI exhibits superior predictive performance compared to TyG in metabolic or cardiovascular diseases (16). A study by Luo et al. (17) showed that there was a u-shaped association between TyG-BMI and 90-, 180-, and 365-day all-cause mortality in critically ill patients with acute myocardial infarction (AMI), which can be used as an effective indicator for early prevention of critically ill patients with AMI. However, relatively few studies have been conducted to assess the correlation between TyG-BMI and all-cause mortality in stroke (16, 18, 19), and the assessment of long-term all-cause mortality, especially in terms of intravenous thrombolysis in elderly patients with AIS, has not been clearly reported. There is a lack of evidence to support the use of the TyG-BMI index as a long-term predictor of all-cause mortality risk in elderly patients with AIS undergoing intravenous thrombolysis. Therefore, the aim of this study was to investigate the correlation and predictive value between TyG-BMI index and all-cause mortality in elderly patients with intravenous thrombolysis for AIS.

## Materials and methods

### Study population

**Figure 1:**
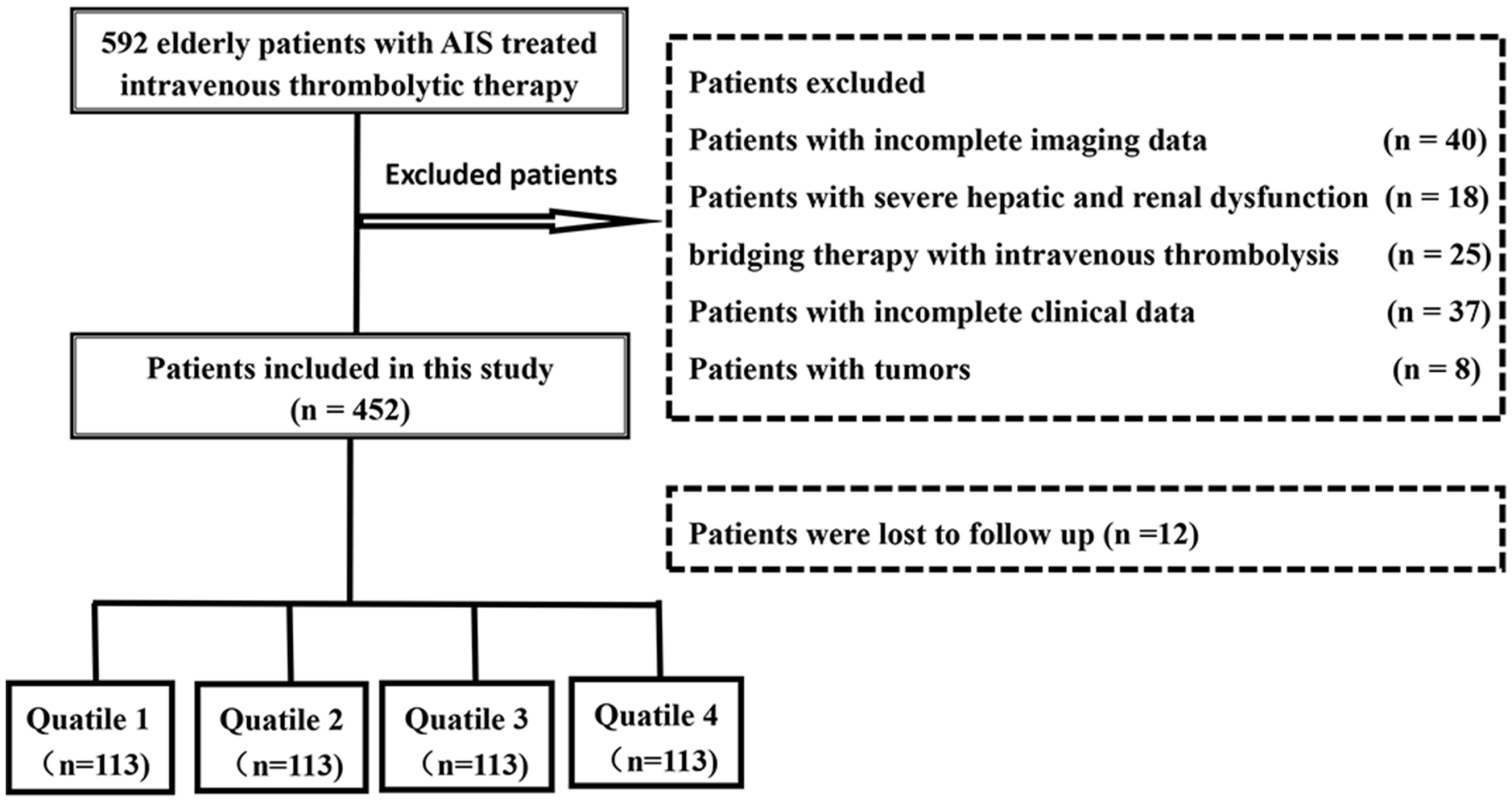
Study flowchart of the present study.

Consecutive patients with acute ischemic stroke who were admitted to The Affiliated Fuyang People’s Hospital of Anhui Medical University and received intravenous thrombolysis from August 2017 to October 2023 were collected, and all patients were ≥60 years old. The exclusion criteria were as follows: (1) incomplete clinical or imaging data, (2) comorbid hematologic-related diseases, severe hepatic and renal dysfunction, etc., (3) patients receiving bridging therapy, and (4) incomplete follow-up data. The study protocol was approved by the hospital ethics committee, and all patients or their relatives signed an informed consent form before inclusion. The detailed methodology of our participant selection process is depicted in Figure 1.

### Data collection

We collect general information about patients, including age, gender, medical history (smoking, alcohol consumption, hypertension, diabetes, dyslipidemia, atrial fibrillation, history of coronary artery disease, history of stroke), blood pressure levels at admission (systolic and diastolic), and clinical characteristics, including laboratory test results at admission, stroke classification, and stroke severity assessed using the National Institutes of Health Stroke Scale (NIHSS) score. According to the acute stroke treatment classification ORG 10172 trial, stroke subtypes are classified into large artery atherosclerosis, cardiac embolism, small vessel occlusion, and others (strokes with other identified causes and strokes with unknown causes). The TyG-BMI index is calculated using the following formula: ln[TG (mg/dL) × FBG (mg/dL)/2] × BMI (20). All laboratory variables and disease severity scores were completed within 24 h of patient admission. Detailed records were kept of the medications prescribed for outpatient treatment, including antiplatelet drugs, anticoagulants, statins, antihypertensive drugs, and antidiabetic drugs.

### Clinical outcome

The primary clinical outcome of this study was all-cause mortality following intravenous thrombolysis in elderly patients with AIS. Enrolled patients with acute cerebral infarction were monitored through telephone interviews or outpatient follow-ups every six months. All-cause mortality refers to the mortality rate from all causes, with a follow-up end date of April 30, 2024. If a patient dies during follow-up, the cause of death is confirmed by reviewing medical records from hospitals and family doctors. All results are reviewed by medical professionals.

### Statistical analysis

Based on the data distribution, continuous variables are expressed as mean ± standard deviation or median (interquartile range), while categorical variables are expressed as proportions. The Kolmogorov–Smirnov test was used to assess the normality of continuous parameters. If continuous variables are normally distributed, t-tests or analysis of variance are used for analysis. If the distribution is non-normal, the Mann–Whitney U test is used. The characteristics of the study participants are expressed using the quartiles of the TyG-BMI index. The Kaplan–Meier survival analysis method is used to assess the incidence of endpoint events between different TyG index level groups, and the log-rank test is used for comparison. The Cox proportional hazards model was used to assess the association between all-cause mortality and potential factors, and hazard ratios (HRs) and 95% confidence intervals (CIs) were calculated.

To evaluate the relationship between different TyG-BMI index and survival status using a combination of univariate and multivariate Cox proportional hazards models. We adjusted for different covariates and constructed three regression models. Model 1: Unadjusted; Model 2: Adjusted for age and gender; Model 3: Adjusted for age, gender, hypertension, diabetes, ischemic heart disease, atrial fibrillation, antiplatelet agents, glucose-lowering agents, NIHSS score, and stroke etiology, using the quartile with the lowest mortality as the reference. In this study, variables with *p* < 0.05 in the univariate regression analysis were included in the multivariate regression analysis. Meanwhile, for variables known to be significantly related to the prognosis of AIS, even if they do not meet the established statistical screening criteria, they are included in the multiple regression analysis model. We also conducted subgroup analyses stratified by potential confounding factors. In addition, we used restricted cubic splines (RCS) to assess the nonlinear relationship between the TyG-BMI index and all-cause mortality. If the relationship was nonlinear, we estimated the critical value by trying all possible values and selecting the most probable tipping point. Then we used two-piece Cox proportional risk models on either side of the inflection point to examine the relationship between TyG-BMI and the risk of all-cause mortality. In addition, C-index, integrated discrimination improvement (IDI), and category-free net reclassification index (NRI).

were used to assess the incremental prognostic value of the TyG-BMI in the final fitted Cox regression models. Data analysis was performed using SPSS version22.0 (SPSS Inc., Chicago, IL, United States) and R version 4.1.2 (The R Project for Statistical Computing, Vienna, Austria). All tests used a two-sided *p*-value of 0.05 as the threshold for statistical significance.

## Results

This study included a total of 452 elderly patients with acute ischemic stroke who underwent intravenous thrombolysis. The median age of the included patients was 73 years (IQR: 67–78), and 264 (58.4%) were male. The mean TyG-BMI index for all included participants was 205.54 ± 38.51. The median follow-up time for all-cause mortality from ischemic stroke was 36.8 months (18.7–52.0 months). During this period, 75 patients (16.6%) experienced all-cause mortality (Table 1).

**Table 1:** Baseline characteristics stratified according to TyG-BMI index quartiles.

Characteristics	Quartile 1	Quartile 2	Quartile 3	Quartile 4	*P*
	(≤177.76)	(177.76–202.66)	(202.66–228.13)	(>228.13)	
Age (years)	74 (69–80)	72 (66–78)	72(67–87)	72(67–87)	0.052
Male, *n* (%)	64 (56.1)	75 (66.4)	58 (51.3)	67 (59.3)	0.141
BMI, kg/m^2^	20.00 ± 1.67	22.84 ± 1.28	24.88 ± 1.53	27.55 ± 2.58	< 0.001
Risk factors, *n* (%)					
Hypertension	78 (69.0)	83 (73.5)	96 (85.0)	95 (84.1)	0.007
Diabetes	14 (12.4)	20 (17.7)	33 (29.2)	52 (46.0)	< 0.001
Dyslipidemia	32 (28.3)	38 (33.6)	45 (39.8)	51 (45.1)	0.050
Ischemic heart disease	18 (15.9)	13 (11.5)	26 (23.0)	29 (25.7)	0.026
Atrial fibrillation	27 (23.9)	17 (15.0)	24 (21.2)	23 (20.4)	0.407
History of stroke	34 (30.1)	35 (31.0)	27 (23.9)	31 (27.4)	0.638
Smoker	39 (34.5)	40 (35.4)	39 (34.5)	32 (28.3)	0.651
Alcohol user	33 (29.2)	41 (36.3)	35 (31.0)	29 (25.7)	0.372
SBP	149.97 ± 20.24	152.12 ± 18.95	152.61 ± 20.83	154.79 ± 21.90	0.371
DBP	83.24 ± 11.52	85.00 ± 11.07	83.05 ± 12.96	84.04 ± 13.72	0.626
Laboratory findings					
TC	4.22 ± 1.02	4.42 ± 1.01	4.65 ± 0.98	4.53 ± 1.16	0.017
TG	0.78 (0.63–0.98)	1.00 (0.76–1.30)	1.26 (0.95–1.64)	1.56 (1.11–2.17)	< 0.001
HDL	1.25 (1.05–1.46)	1.16 (0.99–1.43)	1.10 (0.93–1.25)	1.02 (0.84–1.01)	< 0.001
LDL	2.32 ± 0.81	2.49 ± 0.78	2.69 ± 0.81	2.51 ± 0.89	0.011
FBG	4.99 (4.38–5.83)	5.25 (4.58–5.90)	5.47 (4.83–6.36)	5.90 (5.27–7.70)	< 0.001
TyG	8.09 (7.78–8.31)	8.29 (8.05–8.63)	8.61 (8.30–8.91)	9.01 (8.59–9.49)	< 0.001
TyG-BMI	159.62 ± 12.50	190.65 ± 9.93	215.55 ± 7.16	256.32 ± 25.72	< 0.001
Medications at discharge, *n* (%)					
Antiplatelet agents	99 (87.6)	99 (87.6)	96 (85.0)	100 (88.5)	0.870
Anticoagulants	8 (7.1)	10 (8.8)	12 (10.6)	11 (9.7)	0.816
Statins	112 (99.1)	113 (100.0)	113 (100.0)	111 (98.2)	0.297
Antihypertensives	38 (33.6)	52 (46.0)	62 (54.9)	62 (54.9)	0.003
Glucose-lowering agents	6 (5.3)	11 (9.7)	18 (15.9)	36 (31.9)	< 0.001
Symptomatic steno-occlusion	61 (54.0)	53 (47.3)	49 (43.4)	54 (47.8)	0.455
NIHSS score, median (IQR)	8 (4–13)	6 (3–10)	6 (4–11)	6 (4–11)	0.097
Stroke etiology, *n* (%)					0.715
LAD	43 (38.1)	42 (37.2)	36 (31.9)	40 (35.4)	
CE	22 (19.5)	15 (13.3)	17 (15.0)	15 (13.3)	
SAD	22 (19.5)	21 (18.6)	30 (26.5)	24 (21.1)	
Other and undetermined etiologies	26 (23.0)	35 (31.0)	30 (26.5)	34 (30.1)	
Mortality, *n* (%)	28 (24.8)	8 (7.1)	17 (15.0)	22 (19.5)	0.003

BMI, body mass index; SBP, Systolic blood pressure; DBP, Diastolic blood pressure; TC, total cholesterol; TG, triglyceride; HDL, high-density lipoprotein cholesterol; LDL, low-density lipoprotein cholesterol; FBG, fasting blood glucose; TyG index, triglyceride glucose index; NIHSS, National Institutes of Health Stroke Scale; LAD, large artery disease; CE, cardiac embolism; SAD, small artery disease.

### Baseline characteristics

Baseline characteristics of patients were categorized according to the quartile classification of the TyG-BMI index, as shown in Table 1. The TyG-BMI index level at admission was used to divide patients into four groups [quartile (Q) 1 ≥ 177.76; Q2: 177.76–202.66; Q3: 202.66–228.13; Q4 > 228.13]. Patients in the highest quartile of the TyG-BMI index had higher prevalence rates of hypertension, type 2 diabetes, and coronary heart disease, as well as higher proportions of patients receiving antihypertensive and antidiabetic medications. Additionally, their BMI, triglycerides (TG), total cholesterol (TC), low-density lipoprotein (LDL), fasting blood glucose (FBG), TyG, and TyG-BMI were higher at admission. In contrast, compared with patients in the quartile 4 group, patients in the quartile with lower TyG-BMI index had higher High-Density Lipoprotein (HDL) levels at admission (*p* < 0.05). Meanwhile, all-cause mortality did not decrease with increasing TyG-BMI index, which was lowest in group Q2 and then increased in group Q4.

### All-cause mortality risk analysis

**Figure 2:**
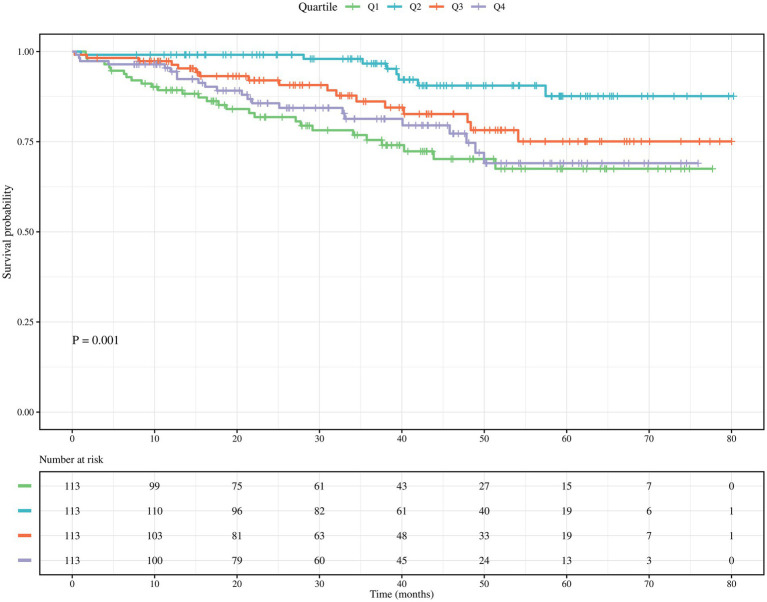
Kaplan-Meier survival curve for all-cause mortality in the elderly patients after intravenous thrombolysis for AIS based on the quartiles of TyG-BMI index.

Kaplan–Meier survival analysis curves were plotted based on the quartiles of the TyG-BMI index to analyze the incidence of the primary outcome between groups. As shown in Figure 2, the all-cause mortality risk of four groups of elderly patients with acute ischemic stroke undergoing intravenous thrombolysis, classified according to the TyG-BMI index, is described. Patients in the higher TyG-BMI groups showed a significantly lower all-cause mortality risk, using the quartile with the lowest mortality as the reference (*p* = 0.001, log-rank test).

To investigate the relationship between TyG-BMI and all-cause mortality in patients, we established three Cox proportional hazards regression models, as detailed in Table 2. We converted TyG-BMI from a continuous variable to a categorical variable based on quartiles. This association was observed in all models. The multivariable-adjusted model showed both the Q1 group [HR, 3.482; 95% CI: 1.560–7.774; *p* = 0.002], Q3 group [HR, 2.819; 95% CI: 1.196–6.640; *p* = 0.018] and the Q4 group [HR, 2.928; 95% CI: 1.259–6.806; *p* = 0.013] were associated with higher all-cause mortality rates, using the quartile with the lowest mortality as the reference.

**Table 2:** Multivariate cox proportional hazards models for the risk of all-cause mortality from acute ischemic stroke.

Variables	Model I		Model II		Model III	
	HR (95% CI)	*P* value	HR (95% CI)	*P* value	HR (95% CI)	*P* value
TyG-BMI						
Q2	Ref		Ref		Ref	
Q1	4.330 (1.972–9.504)	< 0.001	3.540 (1.602–7.824)	0.002	3.482 (1.560–7.774)	0.002
Q3	2.530 (1.091–5.864)	0.030	2.547 (1.095–5.921)	0.030	2.819 (1.196–6.640)	0.018
Q4	3.480 (1.548–7.882)	0.003	3.578 (1.588–8.061)	0.002	2.928 (1.259–6.806)	0.013

Model I: Unadjusted model; Model II: adjusted for gender, age; Model 3: adjusted for gender, age, hypertension, diabetes, Ischemic heart disease, Atrial fibrillation, Antiplatelet agents, glucose-lowering agents, NIHSS score, Stroke etiology. CI, confidence interval; TyG-BMI, triglyceride glucose-body mass index; HR, hazard ratio.

### Restricted cubic spline

**Figure 3:**
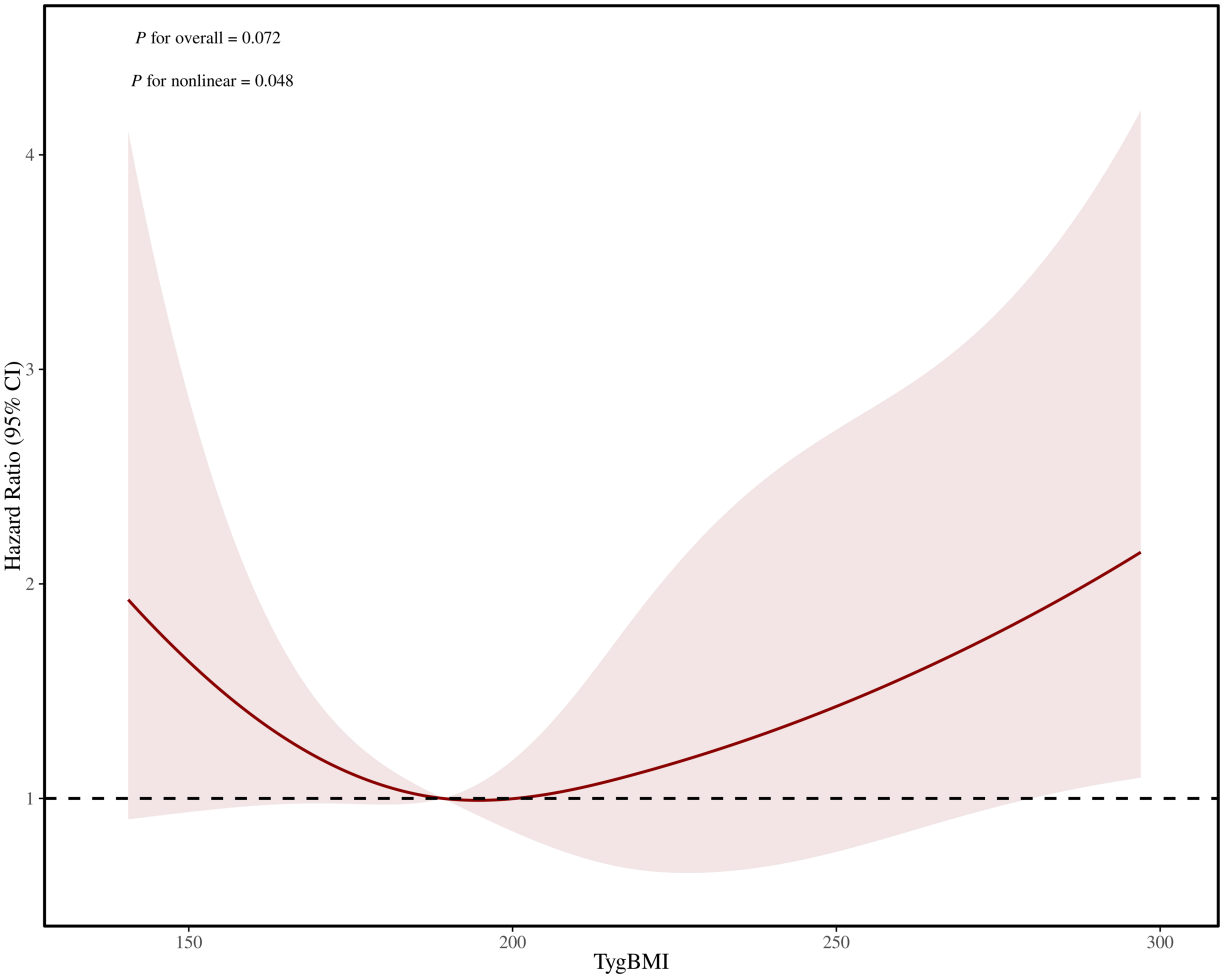
Subgroup analyse of the relationship between TyG-BMI index and all-cause mortality in the elderly patients after intravenous thrombolysis for AIS.

The restricted cubic splines were employed to better characterize and graphically represent the relationship between the TyG-BMI index and all-cause mortality. In a fully adjusted RCS that accounted for confounding factors, the curves showed that there was a nonlinear relationship between the TyG BMI index and all-cause mortality (*p* = 0.048 for non-linearity), with an optimal threshold value of 197.90 for TyG BMI, below the value of 197.90, there was a slight decrease in the mortality rate from all causes (*p* = 0.035). However, above a value of 197.90, all cause mortality slightly increased (HR: 1.007, *p* = 0.089) (Figure 3; Table 3).

**Table 3:** Threshold effect analysis of TyG-BMI on all-cause mortality in elderly patients with AIS undergoing intravenous thrombolysis.

	Adjusted HR (95%CI)	*P*
Fitting by two piecewise cox proportional risk model		
Inflection point	197.90	
<197.90	0.981 (0.963–0.999)	0.035
≥197.90	1.007 (0.999–1.006)	0.089
*P* for likelihood test		0.028

### Incremental predictive value of TyG-BMI

Previous mortality prediction models for AIS with intravenous thrombolytic therapy typically excluded metabolic indicators. The incremental predictive value of TyG-BMI for end points was assessed using the final fitted multivariate Cox regression model component (base model). In the base model, the addition of TyG-BMI increased the predictive value for poor prognosis (C-index:0.725, *p* < 0.001). Furthermore, in the base model, TyG-BMI had significant incremental value in predicting poor prognosis (IDI: 0.109, p < 0.001; NRI: 0.147, *p* = 0.012).

### Stratified analyses

**Figure 4:**
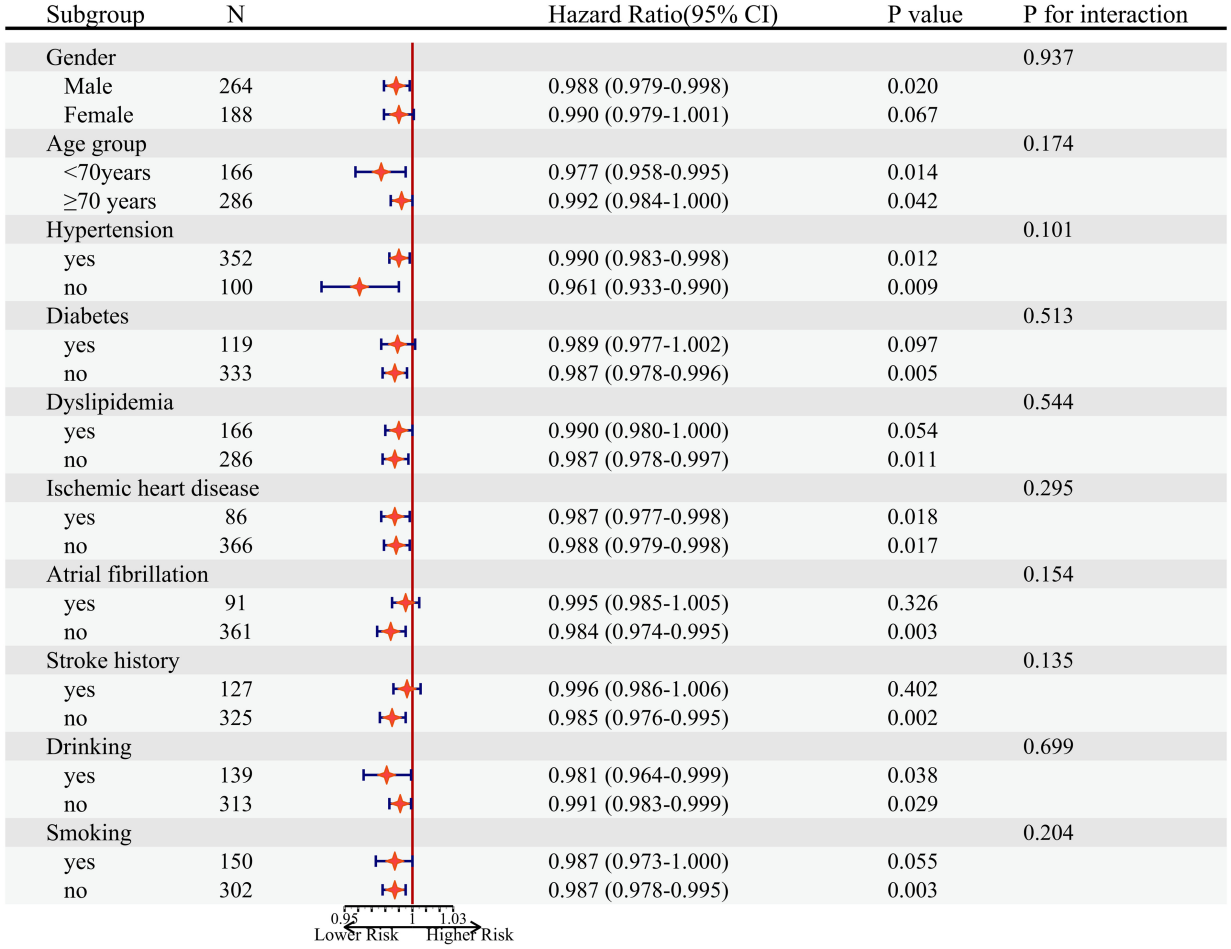
The restricted cubic spline (RCS) analysis between TyG-BMI index and all-cause mortality in the elderly patients after intravenous thrombolysis for AIS.

Stratified analyses were performed to assess the association of TyG-BMI (per SD increment) with risk of all-cause mortality and its subtypes, as showed in Figure 4. No interaction was found between subgroup variables and the association of TyG-BMI with risk of all-cause mortality of AIS.

## Discussion

The purpose of this study was to investigate the relationship between the TyG-BMI index and long-term all-cause mortality in elderly patients with AIS undergoing intravenous thrombolysis, and to identify valuable predictive factors for survival in this patient population. Using Kaplan–Meier survival analysis, univariate and multivariate Cox proportional hazards regression models, and RCS curve analysis, we determined that the TyG-BMI index can serve as a effective biomarker for risk stratification management in elderly AIS patients undergoing intravenous thrombolysis during long-term follow-up. In addition, RCS curve analysis results showed that in both unadjusted and fully adjusted models, the TyG-BMI index was nonlinearly associated with all-cause mortality risk in elderly AIS patients undergoing intravenous thrombolysis, with a threshold close to 197.9. This means that elderly AIS patients undergoing intravenous thrombolysis can achieve a lower risk of death by appropriately increasing their TyG-BMI values. However, by fitting the curve trend in the graph, it can be seen that an excessively high TyG-BMI also increases the risk of all-cause mortality. Therefore, excessively increasing or decreasing the TyG-BMI is not a wise choice. This study demonstrates the importance of TyG-BMI in guiding the development of management strategies for elderly AIS patients undergoing intravenous thrombolysis.

The predictive role of the TyG-BMI index in various diseases has been widely studied. Sun et al. (15) conducted an in-depth study of 1,085 patients with cardiovascular disease (CVD) in the NHANES database from 2007 to 2016, showing that the TyG-BMI index was significantly associated with all-cause mortality in CVD patients. Zhu et al. (21) used Cox proportional hazards regression and restricted cubic spline curves to show that TyG-BMI was significantly associated with all-cause mortality in patients with acute pancreatitis. In critically ill patients with sepsis, TyG-BMI is negatively correlated with mortality at different time intervals. TyG-BMI is a favorable parameter for classifying the risk level of patients with sepsis and predicting their all-cause mortality within one year (22). However, there are still few studies on its relationship with all-cause mortality in stroke patients, and there have been no reports on its relationship with all-cause mortality in elderly AIS patients undergoing intravenous thrombolysis. Our study shows that TyG-BMI can predict long-term all-cause mortality in this patient population. These findings are consistent with those of Huang et al. (19), who used ICU data to investigate the relationship between TyG-BMI and long-term survival in stroke patients. Huang et al. proposed that in severe stroke patients aged 60 years or older, the TyG-BMI index was negatively correlated with all-cause mortality; whereas in severe stroke patients younger than 60 years, the TyG-BMI index was positively correlated with all-cause mortality. Coincidentally, Luo et al. (17) also confirmed a U-shaped association between the TyG-BMI index and all-cause mortality in patients with AMI.

The pathogenesis of AIS patients undergoing intravenous thrombolysis involves a series of complex pathophysiological changes and metabolic disorders, which may cause them to exhibit different risk patterns compared to the general population. In a broader population, elevated TyG-BMI levels are associated with insulin IR-induced glucose metabolism disorders, lipotoxicity, and excessive inflammatory responses, thereby increasing the risk of stroke recurrence (23). Conversely, in stroke survivors, low TyG-BMI levels may lead to malnutrition risk and impaired immune function, and recurrent hypoglycemia may cause irreversible brain damage. These conditions are closely associated with disease severity and increased mortality risk (24, 25). The multiple pathophysiological pathways involved in the TyG-BMI index may explain its association with long-term outcomes in patients with AIS undergoing intravenous thrombolysis. In fact, the link between TyG-BMI and HOMA-IR has been clearly established (26). Therefore, in many studies, TyG-BMI has been advocated as a reliable indicator for assessing IR and its related diseases (16). For example, a cross-sectional study conducted in northern China showed that TyG-BMI demonstrated superiority in detecting IR (27). At the same time, a cross-sectional study investigating health and nutritional status in South Korea found that TyG-BMI can be used as an effective alternative indicator for assessing IR (28).

The mechanisms by which IR causes stroke are multifaceted. First, IR can cause dyslipidemia, high blood pressure, and elevated blood sugar levels. These metabolic abnormalities may accelerate the progression of atherosclerosis, increase the risk of vascular inflammation and thrombosis, and thereby worsen the prognosis of stroke patients (29, 30). In addition, IR may also promote catabolism by enhancing sympathetic nerve activity in muscles, leading to muscle loss and subsequently affecting functional recovery in patients (31). Secondly, the inflammatory response and apoptosis induced by IR play an important role in stroke prognosis (32). In addition, the impact of hyperglycemia, hypertriglyceridemia, and high BMI, which are covered by the TyG-BMI index, on stroke prognosis should not be overlooked. Hyperglycemia can induce oxidative stress by promoting the production of reactive oxygen species (ROS) and reactive nitrogen species (RNS), damaging the blood–brain barrier and exacerbating neuronal damage. Hypertriglyceridemia may directly damage endothelial cells and neurons by promoting vascular inflammation and lipotoxicity, further exacerbating brain damage (33).

Given that some studies have shown that higher TyG-BMI indices are associated with an increased risk of stroke (34, 35), the finding in our study that excessively low TyG-BMI levels were also associated with all-cause mortality appears counterintuitive. However, this study focuses on a specific group of elderly patients who have undergone intravenous thrombolysis after suffering a stroke. The health status and prognosis of this specific group differ significantly from those of the general population who have not suffered a stroke. TyG-BMI is an indicator of IR and metabolic health. In the broader population, higher TyG-BMI levels indicate worsening metabolic health, thereby increasing the risk of cardiovascular events, including stroke. Conversely, in stroke survivors, lower TyG-BMI levels may reflect severe malnutrition or metabolic failure, conditions that are closely associated with disease severity and increased risk of death. This is consistent with previous research findings, indicating that their risk characteristics differ from those of the general population (19).

The advantage of this study lies in the fact that it is the first to apply the TyG-BMI index to assess the risk of all-cause mortality in elderly AIS patients undergoing venous thrombolysis, thereby successfully expanding the clinical applicability of this parameter. Not only does it provide data support for related research, but it also helps establish a more comprehensive secondary prevention system for the long-term prognosis of AIS patients undergoing intravenous thrombolysis. However, this study also has some limitations. First, this study is a single-center study with a small sample size, which makes it difficult to avoid various biases. In the future, more large-sample cohort studies are needed to validate our conclusions. Secondly, like other retrospective cohort studies, this study cannot establish a causal relationship between the TyG-BMI index and all-cause mortality. Third, since we only used some of the data collected as part of routine clinical procedures at admission, we were unable to compare the TyG index with other indicators of insulin resistance, such as the high insulin-normal glucose clamp test and the homeostasis model assessment of insulin resistance (HOMA-IR). Fourth, this study focused on all-cause mortality, cardiovascular mortality and stroke recurrence rates were not included, and we will include these variables in future studies.

## Conclusion

In this study, we found that there was a significant correlation between the TyG-BMI index and long-term all-cause mortality in elderly AIS patients undergoing intravenous thrombolysis. TyG-BMI can serve as a good predictor of long-term all-cause mortality in elderly AIS patients undergoing intravenous thrombolysis.

## Data Availability

The raw data supporting the conclusions of this article will be made available by the authors, without undue reservation.
