# Supplementary material for: Relationship between all-cause mortality and triglyceride-glucose-body mass index in elderly patients with intravenous thrombolysis for acute ischemic stroke: a retrospective cohort study
Source: Front Neurol. 2026 Jan 5;16:1689313. doi: 10.3389/fneur.2025.1689313 (PMC12812639; doi:10.3389/fneur.2025.1689313)
Supplement: Supplementary file 1 [file Table_1.DOCX]

**Table**  Univariate Cox proportional hazards models for the risk of all-cause mortality from acute ischemic stroke

| **Variables** | **Univariate analysis** | |
| --- | --- | --- |
|  | **HR (95% CI)** | ***P value*** |
| Age (years) | 1.070 (1.038-1.103) | < 0.001 |
| Male, *n* (%) |  |  |
| Female | Ref |  |
| Male | 0.936 (0.592-1.479) | 0.776 |
| BMI, Kg/m^2^ | 0.952 (0.888-1.020) | 0.161 |
| **Risk factors, *n* (%)** |  |  |
| Hypertension | 1.137 (0.636-2.034) | 0.664 |
| Diabetes | 1.135 (0.673-1.912) | 0.635 |
| Dyslipidemia | 0.935 (0.578-1.512) | 0.784 |
| Ischemic heart disease | 2.077 (1.270-3.397) | 0.004 |
| [Atrial](file:///D:/%25E4%25B8%258B%25E8%25BD%25BD%25E8%25BD%25AF%25E4%25BB%25B6/%25E6%259C%2589%25E9%2581%2593%25E8%25AF%258D%25E5%2585%25B8/Dict/8.9.8.0/resultui/html/index.html#/javascript:;) [fibrillation](file:///D:/%25E4%25B8%258B%25E8%25BD%25BD%25E8%25BD%25AF%25E4%25BB%25B6/%25E6%259C%2589%25E9%2581%2593%25E8%25AF%258D%25E5%2585%25B8/Dict/8.9.8.0/resultui/html/index.html#/javascript:;) | 3.086 (1.952-4.877) | < 0.001 |
| History of stroke | 1.567 (0.984-2.495) | 0.058 |
| Smoker | 0.776 (0.472-1.276) | 0.317 |
| Alcohol user | 0.681 (0.401-1.157) | 0.155 |
| SBP | 1.004 (0.993-1.015) | 0.498 |
| DBP | 0.997 (0.979-1.016) | 0.791 |
| **Laboratory findings** |  |  |
| TC | 0.868 (0.692-1.088) | 0.219 |
| TG | 1.156 (0.913-1.463) | 0.228 |
| HDL | 1.006 (0.455-2.225) | 0.987 |
| LDL | 0.829 (0.622-1.106) | 0.203 |
| FBG | 1.136 (1.053-1.225) | 0.001 |
| TyG | 1.309 (0.936-1.831) | 0.116 |
| TyG-BMI | 1.005 (0.999-1.010) | 0.114 |
| TyG-BMI, Quartiles |  |  |
| Q2 | Ref |  |
| Q1 | 4.330 (1.972-9.504) | < 0.001 |
| Q3 | 2.530 (1.091-5.864) | 0.030 |
| Q4 | 3.480 (1.548-7.882) | 0.003 |
| **Medications at discharge, *n* (%)** |  |  |
| **Antithrombotic drugs** |  |  |
| Antiplatelet agents | 0.499 (0.287-0.867) | 0.014 |
| Anticoagulants | 1.400 (0.698-2.811) | 0.343 |
| Statins | 0.340 (0.047-2.458) | 0.285 |
| Antihypertensives | 1.231 (0.782-1.938) | 0.370 |
| Glucose-lowering agents | 1.009 (0.518-1.966) | 0.979 |
| Symptomatic steno-occlusion | 1.519 (0.961-2.401) | 0.073 |
| NIHSS score | 1.092 (1.058-1.126) | < 0.001 |
| **Stroke etiology** |  |  |
| SAD | Ref |  |
| LAD | 1.653 (0.818-3.342) | 0.162 |
| CE | 4.447 (2.233-8.858) | < 0.001 |
| Other and undetermined etiologies | 1.784 (0.844-3.771) | 0.130 |

BMI, body mass index; SBP, Systolic blood pressure; DBP, Diastolic blood pressure; TC, total cholesterol; TG, triglyceride; HDL, high-density lipoprotein cholesterol; LDL, low-density lipoprotein cholesterol; FBG, fasting blood glucose; TyG index, triglyceride glucose index; NIHSS, National Institutes of Health Stroke Scale; LAD, large artery disease; CE, cardiac embolism; SAD, small artery disease.
